# Mapping Post-Glacial expansions: The Peopling of Southwest Asia

**DOI:** 10.1038/srep40338

**Published:** 2017-01-06

**Authors:** Daniel E. Platt, Marc Haber, Magda Bou Dagher-Kharrat, Bouchra Douaihy, Georges Khazen, Maziar Ashrafian Bonab, Angélique Salloum, Francis Mouzaya, Donata Luiselli, Chris Tyler-Smith, Colin Renfrew, Elizabeth Matisoo-Smith, Pierre A. Zalloua

**Affiliations:** 1 Computational Biology Center, IBM TJ Watson Research Centre, Yorktown Hgts, NY, USA; 2 The Lebanese American University, Chouran, Beirut, Lebanon; 3 The Wellcome Trust Sanger Institute, Wellcome Trust Genome Campus, Hinxton, UK; 4 Département Sciences de la Vie et de la Terre, Université Saint-Joseph, Mkalles, Lebanon; 5 University of Portsmouth, School of Biological Sciences, Portsmouth PO1 2DY, UK; 6 Department of Evolutionary Experimental Biology, Via Selmi, 340126 Bologna, Italy; 7 McDonald Institute for Archaeological Research, University of Cambridge, CB2 3ER, UK; 8 Department of Anatomy and Allan Wilson Centre for Molecular Ecology and Evolution, University of Otago, Dunedin, New Zealand; 9 Harvard School of Public Health, Boston, MA, USA

## Abstract

Archaeological, palaeontological and geological evidence shows that post-glacial warming released human populations from their various climate-bound refugia. Yet specific connections between these refugia and the timing and routes of post-glacial migrations that ultimately established modern patterns of genetic variation remain elusive. Here, we use Y-chromosome markers combined with autosomal data to reconstruct population expansions from regional refugia in Southwest Asia. Populations from three regions in particular possess distinctive autosomal genetic signatures indicative of likely refugia: one, in the north, centered around the eastern coast of the Black Sea, the second, with a more Levantine focus, and the third in the southern Arabian Peninsula. Modern populations from these three regions carry the widest diversity and may indeed represent the most likely descendants of the populations responsible for the Neolithic cultures of Southwest Asia. We reveal the distinct and datable expansion routes of populations from these three refugia throughout Southwest Asia and into Europe and North Africa and discuss the possible correlations of these migrations to various cultural and climatic events evident in the archaeological record of the past 15,000 years.

Climate shifts dramatically influenced the occupation of Southwest Asia by anatomically modern humans since their first occupation at the sites of Skhul and Qafzeh in Israel some 80 to 130 ka^1^. These first archaeologically identified modern humans outside of Africa appear to have left no genetic impact, with the next modern human remains in the Levant dating to around 45–50 ka^1^. Climate change impacts on population mobility and cultural developments have been hotly debated^2^. Aridity restricted larger human populations to refugia sited around the Mediterranean^3^, the Black Sea^4^, and possibly the southern Arabian Peninsula^5^, that were distinguished by tool cultures in the archaeological record^6^,^7^, during the Last Glacial Period (LGP) ending some 12 ka. Post-glacial warming, and later, agriculture, allowed expansions of these populations within SW Asia^8^. Archaeological evidence shows post-LGP population expansions shifting from mobile foraging to a more sedentary lifestyle^4^,^9^ with permanent settlements, early cultivation of wild plants^10^,^11^, stored goods, and emerging trade and exchange networks^12^,^13^. The Fertile Crescent based “Neolithic Revolution” replaced most cultures across the region, spreading Indo-European languages from an Anatolian homeland^14^, both westward to Europe, northward to the steppes, and eastward to the Iranian plateau and beyond.

Autosomal analyses have identified a cryptic population, “Basal Eurasians,” that injected significant genetics into the European population distinct from the Southwest Asian agricultural revolution signature^15^. Subsequent work^16^,^17^ has suggested that Yamnaya aDNA appears to have been a source of the genetics associated with corded ware culture, and provides further evidence for a late Neolithic through early Bronze Age wave introducing Indo-European languages.

Lineages that evolved in relatively isolated refugia populations could carry genetic evidence of that isolation and subsequent expansion. Such signals identified source populations for admixture events marking European settlement^15^,^16^,^18^,^19^. However, specific associations between Southwest Asian post-LGP expansion genetics and their original refugia, and the identification of the timing and directions of their various dispersal events, are still being determined. Most recently, analyses of complete mitochondrial genomes have identified a possible Arabian refugia in the LGP, within which several mtDNA haplogroups including R0a emerged and from which they dispersed to the Fertile Crescent, the Levant and the Horn of Africa^20^. Y-chromosome analysis has identified highest frequencies for J1 haplogroup to be most common in the Saudi Peninsula^21^ marking the Muslim expansion^22^ with J2 being common in the coastal Levant^23^, and identified early on as a possible marker of the European expansion of the post-Neolithic expansion^8^,^24^, while their origins have been identified roughly within Iran, Armenia, Georgia, and/or Eastern Turkey. However, even though J2 appeared to mark a post-Neolithic expansion, this haplogroup has been identified in ancient DNA analysis of remains archaeologically associated with the late Bronze Era^25^. Both Haplogroups origins have been identified roughly within Iran, Armenia, Georgia, and/or Eastern Turkey.

Here, we take the approach of identifying and dating population isolation prior to expansion, and tracing routes of dispersal throughout Southwest Asia and into Europe and Africa. We analyzed a comprehensive autosomal and Y-chromosome dataset of Eurasian and African populations identifying genetic signals of regional LGP population isolation, and contrasted expansion time estimates and dispersal routes in the region with archaeological^4^,^9^, palaeontological^26^, palaeobotanical^27^, and climate^4^,^27^ data.

## Material and Methods

### Samples and Genotyping

A total of 8,515 samples were analyzed (Supplementary Table S1) for their Y-chromosome genetics. All participants recruited and genotyped by our team had at least three generations of paternal ancestry in their country of birth and provided details of their geographical origin. A written informed consent was signed and obtained by each participant prior to recruitment for this study. The study protocol and the informed consent form were approved by the IRB of the Lebanese American University. The study methods were carried out in accordance with the principles of the Declaration of Helsinki. DNA was extracted from blood or buccal swabs using a standard phenol–chloroform protocol. Samples were genotyped for binary Y chromosome polymorphisms as reported previously^23^.

Our previously described samples (n = 2047)^23^,^28^,^29^ were further subtyped to achieve the same Haplogroup (Hg) differentiation as the new samples analyzed here^23^,^28^,^29^, with the additional 6 SNPs (J1e-P58, J2a-M410, J2b-M12, E1b1b1a-M78, E1b1b1b-M81 and E1b1b1c-M123). DNA samples were also typed for 11 microsatellite loci (DYS 388, 389I, 390, 391, 393, 19′, 437, 439, 389II, 392 and 438).

In addition, Y- Hg and haplotype data were also incorporated from prior studies (Supplementary Table S1), including 727 newly genotyped samples from Armenia (ARM - 402 samples), Armenians originating in SE Turkey (TUR - 126), Bahrain (BAH - 40), Iraq (IRQ - 70), the Kingdom of Saudi Arabia (KSA - 7), Cyprus (CYP - 38) and Libya (LIB - 44) (Study references included in the supplementary information.) Haplogroup markers employed by the studies were catalogued (Supplementary Table S2). The Y-STR Haplotype Reference Database (YHRD) tree and International Society of Genetic Genealogy (ISOGG 2011) tree were compared, with YHRD being used as the base nomenclature (Supplementary Table S2) for construction of most derived sets.

Autosomal data were obtained from a prior study^30^, which had selected 75 Lebanese samples from a pool of 1,341 by stratified random sampling. That dataset had included 994 samples from 48 populations spanning SW Asia, Europe, N. Africa, and into S. Asia. All of these samples were analyzed using Illumina 610 K or 660 K bead arrays. The results were filtered requiring 99% genotype success rate, and removal of sex-linked and mtDNA SNPs, yielding 505,859 SNPs. Further LD pruning (excluding r2 > 0.4) yielded 244,919 SNPs. These QC filtering steps were performed with PLINK^31^. From these, we retained 174 samples representing SW Asia populations.

### Population Pooling

The initial populations were defined primarily based on modern national boundaries or sub-regions. Pooled populations were identified through preliminary BATWING (see Supplementary information) analysis as described below.

We sought to analyze the data without preconceptions of the history of southwest Asian populations, allowing the data to govern their own analysis. Our data preparations sought to ensure the STR loci and haplogroup derivations were uniform across populations and haplogroups, requiring factoring to a lowest common denominator across all haplogroup and population studies.

BATWING analyzes population splits of the form *((A,B),(C,D))* as two topologies instead of one, counting an *(A,B)* split prior vs. subsequent to the *(C,D)* split as two distinct topologies, inducing a spurious interaction between *(A,B)* and *(C,D)* split times, even though those splitting events are independent. Running BATWING with two conformations *(AB, (C,D))*, and *((A,B), CD)*, where *AB* represents *A* and *B* pooled, and *CD* represents *C* and *D* pooled removes the interaction. The two estimates of the first split provide a check for consistency. This process also establishes pooled populations. We adapted the pooled regions for the second phase of analysis when pooling of similar regions following similar expansion routes were shared by multiple haplogroups. The resulting pooled regional designations are Arabia (Saudi Arabia, Emirates, Qatar, Bahrain), Yemen (by itself), Armenia and Turkey (one region), Caucasus (Georgia), Mesopotamia (Iran, Iraq, Kuwait), Cyprus, Southern Levant (Jordan, Palestine), Northern Levant (Lebanon, Syria), Egypt, North Africa (Libya, Morocco, Tunisia, Algeria), and Ethiopia (the data from this preliminary analysis is not shown).

Since BATWING input only involved genetic data and population assignments, results might have been geographically random. Instead, the modal tree population splits easily lie on a map. The combinatorial enumeration of trees vs geography is non-trivial. However, simple limits may be estimated by considering the chances that an east-west split would have accurately classified Northern and Southern Levant (4 populations) on one side and Mesopotamia and Arabia (7 populations) by chance on the other, for example, which has a p-value = 0.003 by a Fisher exact test.

STR-based *F*_*ST*_ computations, MDS, and complete linkage agglomerative clustering were not sufficient to resolve regions in a meaningful way. AMOVA (see supplementary information) also did not show significant regional organization. Haplogroup J1′s *F*_*CT*_ = 0, with p-value = 0.097 that a random construction would be larger. Haplogroup J2′s *F*_*CT*_ = 0.0097, with p-value = 0.486. Haplogroup E1b1b1 shows the greatest divergences, with *F*_*CT*_ = 0.233, with p-value = 0.00067.

The parameter set for BATWING is described in the supplementary information. Error bar 95% CIs about the median range from 1/1.75 to ½ of the median to 1.75 to 2 times the median.

### Analysis

#### Frequency and variance maps

Maps showing haplogroup frequency distributions (Supplementary Figure S1) were derived from data from our laboratory listed in Table S1. Frequency (Supplementary Figure S2) and variance (Supplementary Figure S3) contour map construction is described in the Supplementary information.

### PCA and MDS

Principal component analysis (PCA) was applied to relative haplogroup frequencies. Multidimensional scaling (MDS), was applied to *R*_*ST*_ distances on STR loci across haplogroups from the full derived set, as well as within J2 and J*(xJ2) haplogroups. Details on these methods are in the Supplementary information.

### Network

NETWORK was used to compute Reduced Median (RM) networks (Supplementary information) for haplogroups J1e, J*(xJ1e), J2a, J2b, and J2.

### Y TMRCA Calculation

BATWING parameters are described in the supplementary information. Times of Most Recent Common Ancestor (TMRCA) estimated using UEP time estimation on J subhaplogroups, J1, J1e, J2, J2a1, J2a1, J2a2, J2a2a, and J2b, for whole-population data drawn from Armenians, Caucasians, Iranians, Jordanians, Lebanese, Palestinians, South East Turks, and Turks, Syrians and Africans are listed in Table 1.

### Y Haplogroup expansions

BATWING population splitting was applied to individual haplogroups drawn from both whole-population and haplogroup-specific studies. BATWING samples STR haplotype phylogenies and population split phylogenies, and Bayesian prior distributions to estimate effective population sizes, and mutation rates, using a Metropolis-Hastings Markov-Chain Monte-Carlo simulation. BATWING assumes a single-step mutation model, and coalescence governed by an effective population size, and it models population splitting by dividing the total parent population effective population size among the child populations. The population splitting assumption is the weakest link in application to individual haplogroups. BATWING provides a fixed then expanding effective population size model, allowing for substantial flexibility in population coalescences that the expanding haplogroups will have evolved within as they migrated. Detailed considerations are offered in the supplementary information.

### Genome-wide samples and analysis

We analyzed 174 samples from 9 Southwest Asian populations (Georgians, Armenians, Turks, Lebanese, Syrians, Palestinians, Jordanians, Saudis and Yemenis) using published genome-wide marker data^29^,^30^,^32^. A PCA removing outliers left 155 samples (supplementary information). ADMIXTURE (supplementary information) was applied to the reduced set to identify ancestral populations and to compute ancestral *F*_*ST*_s. Divergence dates were estimated from *F*_*ST*_ estimates (see the supplementary information for details).

We tested whether or not the populations were genetically differentiated. Pair-wise *F*_*ST*_s were computed^33^ between all individual samples (distinct from the ancestral *F*_*ST*_s described above). MDS was also applied to the pairwise *F*_*ST*_’s. We selected a number of MDS dimensions large enough so that the histograms of reconstructed distances became stable. Agglomerative clustering using Ward’s method and Neighbor-Joining (NJ) was applied to the distances reconstructed from the three leading dimensions produced by MDS (supplementary information). While agglomerative clustering does not model population dynamics or events, *F*_*ST*_s are related to divergence times through coalescence, mutation, and migration. This provided a visual test of geographic organization of pair-wise *F*_*ST*_ based genetic signatures. We tested whether the geographical/population associations could have occurred by chance. Mantel distance correlation tests (supplementary information) contrasted first, the pairwise distances computed from the leading PCA components, and second, *F*_*ST*_’s, with a population similarity matrix (distance of 0 for same populations, 1 if different) according to the individuals’ population assignments.

## Results

**Figure 1:**
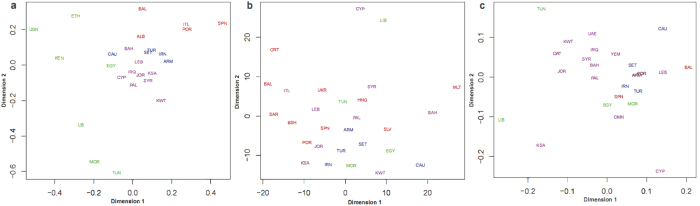
MDS analyses of *R*_*ST*_ distances based on STR haplogroup variances (**a**) across all haplogroups, for (**b**) J*(xJ2), and (**c**) J2.

Y-chromosome haplogroups J1 and J2 and E1b1b are the major paternal lineages, accounting for 50.9% in Southwest Asia and North Africa. The Y haplogroup frequency pie-chart map (Supplementary Figure S1), and PCA (Supplementary Figure S4) both show broad geographic differentiation. Similarly, an MDS plot based on Y-STR across all haplogroups (Fig. 1a) reveals similar geographic organization, reflecting correlation between STR haplotypes and haplogroups. However, the MDS shows weak geographical organization among J2 and J*(xJ2) haplogroup Y-STR *R*_*ST*_ distances analyses compared to *R*_*ST*_ distances analyses applied to all haplogroups (Fig. 1b and c). Likewise, a reduced median NETWORK analysis of STR haplotypes within J haplogroups, namely J2d, J1e and J1xJ1e, also reveals less geographical organization (Supplementary Figure S5). Since Y-chromosomal lineages generally show strong geographical structure, the lack of such structure within J is unexpected.

Maximal diversities, indicative of possible origins, are observed for J*(xJ2) in the Caucasus and for J2 in Armenia, decreasing south and east, although high diversities do not entirely correlate with high relative frequencies (frequency contour Supplementary Figure S2 and variance contour Supplementary Figure S3). BATWING TMRCA (Supplementary information) estimates for J*, J1, J1e, J2, and E1b1b are shown in Table 1. These results indicate slightly greater time depth information of J, J1, and J2 in Turkey and populations from the Caucasus, in agreement with their higher diversity in this area. They also show greater differentiation of the E1b1b1 haplogroups between North Africa and the rest of Asia. The great time depth of J2 and E in Iberia are consistent with a longer period of isolated evolution. Haplogroup J* is the least differentiated in time, but shows maximum values in Turkey Iran as well as North Africa, with decreasing values progressively further away from these geographical locations. Due to the phylogenetic relationship between J1 and J2 markers, the TMRCAs for these are identical. The J1 and J2 split shows deepest time in the Caucasus, Syria and Turkey at 8.9 ka and 8.4 ka, respectively.

**Figure 2:**
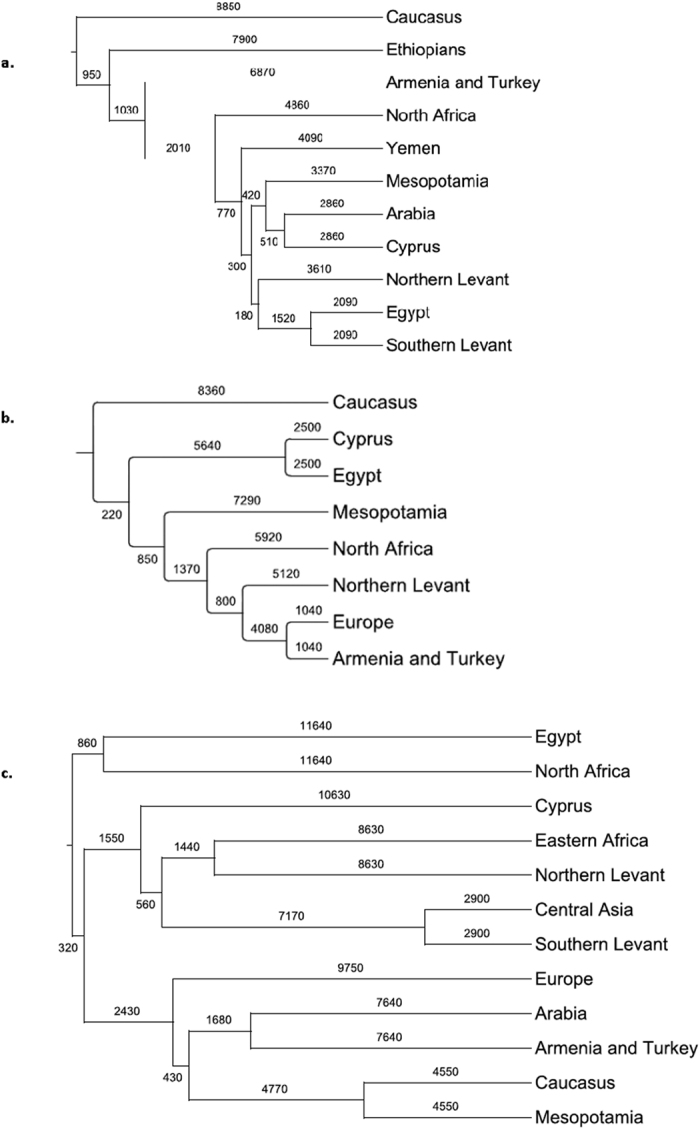
BATWING population split time estimates for Middle Eastern populations marking expansion histories for haplogroups (**a**) J1, (**b**) J2 and (**c**) E1b1b1.

The population split estimates (Fig. 2) show divergences largely reflecting the trend in TMRCAs with older dates closer to the Caucasus, but with differentiation times roughly 1/3 of the TMRCAs, suggesting that 2/3 of STR diversity evolution occurred prior to the earliest population differentiations that followed the Late Glacial Period expansions. Expansions therefore distributed a broad spectrum of shared STR haplotypes that had already evolved within their isolated populations. This broadly shared diversity explains the weak geographical discrimination observed in the phylogenetic networks (Supplementary Figure S5) and the lack of organization found in the MDS analysis for haplogroup specific J*(xJ2) and J2 Y STR *F*_*ST*_s (Fig. 1b and c).

The J1 haplogroup shows greatest differentiation of the Caucasus at 8.9 ka, then Ethiopia at 7.9 ka, followed by Armenia and Turkey at 6.9 ka. The J2 Haplogroup shows an earliest isolation of the Caucasus at 8.4 ka. For the E1b1b1 haplogroup, Egypt, North Africa, and the rest of the populations differentiated at 11.6 ka nearly polytomously (Fig. 2).

**Figure 3:**
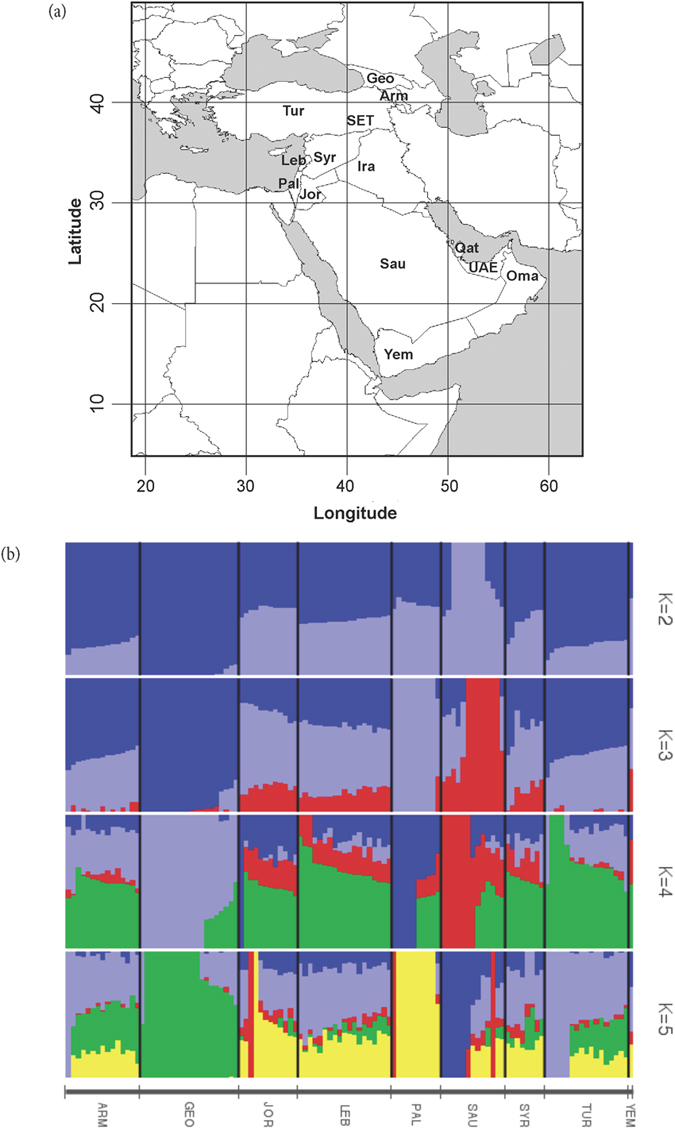
Genome-wide analysis of populations from Southwest Asia using 188,974 autosomal SNPs. (**a**) Map showing populations analyzed (Base map constructed from Map data: Google, Digital Globe, https://earth.google.com); (**b**) Population. structure inferred by ADMIXTURE. Each horizontal line represents ancestry probabilities of an individual in 2 constructed ancestral populations.

**Figure 4:**
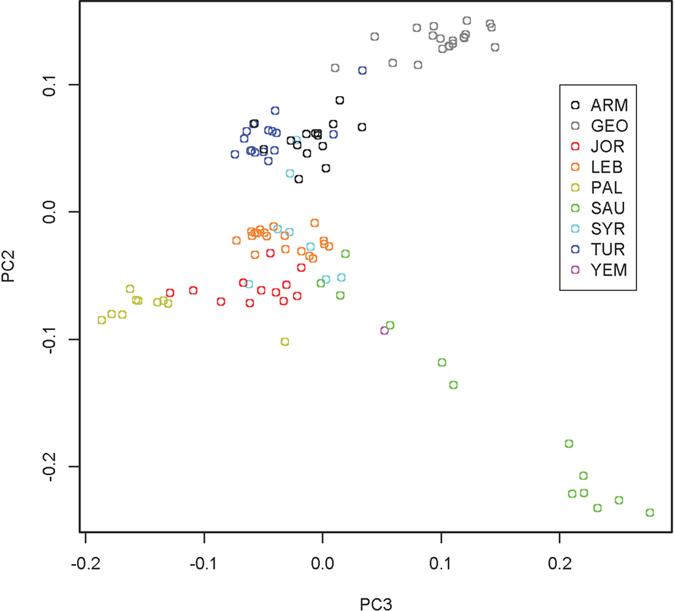
PCA showing Genome-wide analysis of populations from Southwest Asia using 188,974 autosomal SNPs. Eigenvalues were 3.76 × 10^5^ for PC1 (not plotted, reported for scale), 2.16 × 10^5^ for PC2, and 1.62 × 10^5^ for PC3.

We performed an ADMIXTURE analysis to identify and quantify ancestral components in Southwest Asians across all chromosomes using 188,974 SNPs (Fig. 3). ADMIXTURE’s cross-validation (CV) shows decreasing CV error with decreasing dimensions to *K* = 1, with a basin from *K* = 3 to *K* = 1. In *K* = 3, Saudi, Georgian, and Palestinian populations show samples marking pure derivations of the *K* = 3 ancestral populations. Since the cross-validation scores minimized at K = 1, its utility in identifying a best estimate for ancestral population count is weak for these populations. However, the PCA and pairwise *F*_*ST*_ results give a strong suggestion that more ancestral signals are present. For this reason, ADMIXTURE analyses were presented for higher *K*s in order to offer information regarding the structure PCA presents, showing results consistent with PCA and pairwise *F*_*ST*_. The rest show mixtures of these three, with Northern and Palestinian ancestral contributions appearing in Levantine and Fertile Crescent regions including Turkey and Armenia. The split to *K* = 4 primarily impacted the Palestinian ancestral group, splitting it. That 4^th^ ancestral group is not purely represented in any of the modern populations, but is mostly represented in the Levantine through the Fertile Crescent regions. However, some of the samples clearly shifted character from mixtures to pure ancestral groups, reflecting increasing instability in the computations. PCA analysis (Fig. 4) most clearly identifies the same three evolutionary centers as extremes in the plot, strongly echoing, and largely clarifying, the information contained in the ADMIXTURE analysis. Specifically, the *K* = 5 ADMIXTURE identifies an ancestral population that completely dominates the Palestinian population, which corresponds to the Palestinian group on the PCA. The Saudi Arabian population shows an ancestral population marked in red for *K* = 5, with admixture into the Levantine populations, but nearly absent in Turkey, Armenia, and totally absent from Georgia. In the PCA, the Saudi population occupies a well-defined area, with a tail that admixes with the Levantine populations. The Georgian population is dominated by a strongly defined ancestral population, which shows admixed presence along the northern Fertile Crescent, and into the Levant. This is consistent with the genetic distances as shown in the PCA. The ADMIXTURE *F*_*ST*_’s tended to increase with increasing K. The largest *F*_*ST*_ in the K = 3 group was 0.075, and the smallest was 0.057. This corresponds to 36.4 ka and 27.4 ka (we assume *N*_*e*_ = 8,060 - the harmonic average of 7,000 and 9,500 as in the supplementary information, which yields 1260 generations, or 36.4 ka for *F*_*ST*_ = 0.075, and 946 generations or 27.4 ka for *F*_*ST*_ = 0.057), all well into the LGP.

The Ward and NJ cluster results for pairwise *F*_*ST*_s (Supplementary Figure S6) show the northern populations tending to isolate toward one end of the cluster, and southern populations isolating toward the other end, with Levantine and others in the middle, showing substantial differentiation between these branches. Mantel tests comparing geography to *F*_*ST*_for pairwise samples showed p-values < 10^−6^ given 10^6^ iterations.

The unrooted NJ trees show strikingly varying branch lengths, and remarkably differentiated substructure within the major putative northern and southern refugia, as well as some suggestion of a Levantine refugium. For those populations expanding from the north, the trees reveal a similar east/west split as identified for the J1/J2 expansions. We note the pairwise *F*_*ST*_ date estimates are much younger, at 6.6 ka (assuming initial divergence around 11 ka) than the ancestral differentiation dates suggested by the ADMIXTURE derived *F*_*ST*_ ancestral date estimates of 37.4 ka and 27.4 ka.

## Discussion

**Figure 5:**
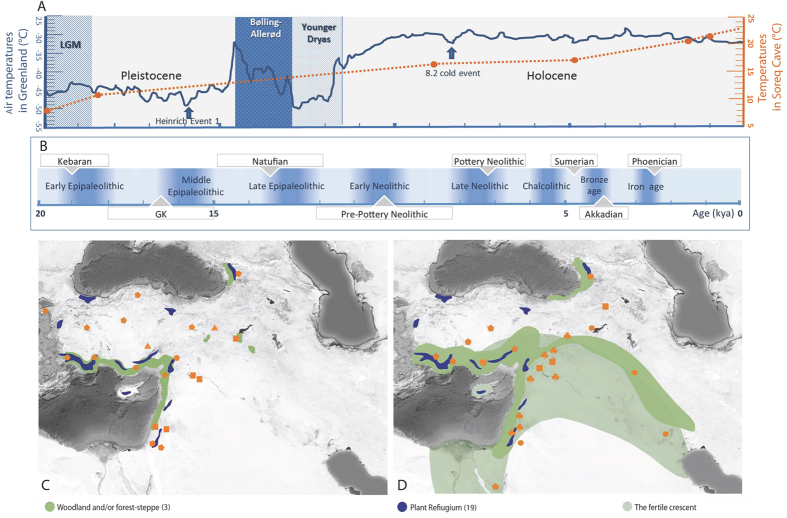
(**A**) Reconstructed air temperatures from the GISP 2 Ice core in Greenland (blue solid line); Isotope values from Soreq cave, Israel (orange dotted line). (**B**) Chronology of cultural entities in the Levant. (**C**) Archaeological evidence of human activities in the Upper Paleolithic; 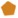 human occupation signs 29–15.2 ka, 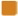 wood charcoal, nuts 15.9–11.2 ka, ▲centers of obsidian trade 16–14 ka. (**D**) Evidence of human activities in the Neolithic; 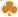 plant domestication 12.5–9.6 ka, 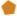 human occupation signs 11.9–5 ka, 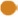 animal domestication, 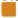 charred plants, agriculture and charcoal 10.2–7.3 ka. (Base map constructed from Map data: Google, Digital Globe, https://earth.google.com).

Our Y chromosome and autosomal analysis identified genetic signatures of three likely centers of isolated evolution followed by population expansions. These modern centers correspond to archaeologically known LGP refugia^4^,^9^,^27^ in Southwest Asia^4^,^5^,^34^. The first, identified by Obsidian sourcing^35^ as an expansion center in Georgia/eastern Turkey^12^,^13^. Clear evidence of trade, and tool cultures mark the second refuge in the northern Levant^7^. The third in the southern Arabian Peninsula^5^ identified recently by Gandini *et al*.^20^. Archaeologically, the time period marking post glacial population expansions through Southwest Asia is associated with the early and middle Pre-Pottery Neolithic B, the increased reliance on domesticated plants and animals, increased evidence of trade and exchange along the earlier established obsidian trade routes, and coastal to inland trade of marine resources^36^ (Fig. 5).

BATWING characterized three features in the genetic record during the expansion of J1 and J2. The earliest dates, with the oldest differentiations for J2 at 8.4 ka, and J1 at 8.9 ka, show an early divergence between the Caucasus from the rest of the populations. BATWING does not model admixture; population split estimates dates to the time admixture ceases. In practice, very minor admixture can cause split times to be underestimated. The branches, to the east down through Mesopotamia and to the west down through the Levant, maintained mutual isolation. Those earliest dispersions show post-last-glacial-period dates but could have been earlier than BATWING estimated if admixture persisted. Later, a significant number of branches occurred within a very narrow window of time, nearly polytomously, followed by relatively stability, marking the emergence of stronger regional isolation suggesting sedentism. That window marks a period near the end of the Holocene Climatic Optimum (HCO - 9 ka–5 ka). Generally, the expansions show movement from northern regions until the HCO, with subsequent isolation and differentiation in the arable regions (Fertile Crescent, Levant, etc.) during aridification.

Subsequent expansions of J1 show the Mesopotamian branch extending to Arabia and Yemen at about 4 ka. The signal of the original North African J1 expansion is overwhelmed in Egypt by more recent Levantine J1s (Fig. 2a). J2 shows similar but deeper isolation during the HCO, with a similar expansion date into North Africa (5.9 ka) as J1 (4.9 ka, Fig. 2b). Yet, its isolation in Egypt is far older, around 8.1 ka. Further, J2′s Mesopotamian population differentiated earlier from the Levantine populations (7.3 ka), with much more recent admixture among J2′s in Turkey (5.1 ka).

Y-M35 (E1b1b1) dominates North Africa much more than Southwest Asia, with a Neolithic split at 11.6 ka between eastern and western North Africa. BATWING will interpret unique haplotypes among rare E1b1b1 samples as older isolation. Most of the populations show apparent isolation dating to the HCO with some associations showing as recent links between central Asians and the Southern Levant (2.9 ka), and the Caucasus with Mesopotamia (4.6 ka Fig. 2).

The population split estimates (Fig. 2) show divergences largely reflecting the trend in TMRCAs with older dates closer to the Caucasus, but with differentiation times that are roughly 1/3 of the TMRCAs, suggesting that 2/3 of STR diversity evolution occurred prior to the earliest population differentiations that followed the Late Glacial Period expansions. Expansions, therefore, distributed a broad spectrum of STR haplotypes that had already evolved within their refugia. This diversity explains the weak geographical discrimination observed in the phylogenetic networks (Figure S5) and the lack of organization found in the MDS analysis for haplogroup specific J*(xJ2) and J2 Y STR *F*_*ST*_s (Fig. 1b and c).

Our autosomal data analyses, PCA and ADMIXTURE, and MDS and Neighbor-Joining cluster analysis applied to individual *F*_*ST*_s, suggest expansions from three centers dominated the peopling of Southwest Asia: an expansion from an ancestral northern population consistent with the Y-chromosome derived Georgian refugia, a southern expansion from a refugium in the Arabian Peninsula, and a Levantine center. Though the leading two PCA components of a prior analysis including European data showed a linear extension from Turkey to the Saudi peninsula, it did not identify a Levantine ancestral population^15^. More surprising, our Y-chromosome BATWING analysis did not even reveal the existence of ancestral expansion from Arabian Peninsula or the Levantine region. A previous study also noted that Saudi Y genetics derived from elsewhere^21^. Today, the J1 haplogroup dominates the Arabian Peninsula region (Supplementary Figure S2), though its greatest diversity (Supplementary Figure S3), marking its origin, is seen in the north. Meanwhile, the J2 diversity contour maps suggests centers from the Levant and Tarsus mountains. The strong coastal vs. inland distributions of these two haplogroups^23^,^37^ belies the proximity of J1 and J2 origins.

The Levantine autosomal expansion appears, to parallel the Natufian expansion (10 ka according to pairwise-*F*_*ST*_ numbers)^7^ and the BATWING dates marking regional differentiations are similar to the pairwise *F*_*ST*_ derived dates. The larger scale ADMIXTURE results however, correspond more closely with the earlier isolations observed in Y STR estimates of African population J and E (e.g. E-M81 is estimated at 14.2 kya reported at YTree https://www.yfull.com/tree/E-M81/) haplogroups. This contrast suggests ongoing mobility and admixture within Southwest Asia subsequent to the expansion into Africa, since BATWING identifies splitting times since the end of admixture, or else the BATWING dates within Southwest Asia refer to a more recent expansion that completely subsumed an earlier expansion that reached into Africa. This mobility and admixture was greatly reduced following post HCO agricultural settlement.

The Y-chromosomal and autosomal ancestral reconstructions from modern populations in Southwest Asia and Europe presented here seem to agree with other evidence of ancient expansions. However, this contradicts more recent aDNA autosomal analysis described below.

A recent study^15^ incorporating aDNA data, along with modern population structure, inferred that most Europeans derive from three primary population groups. One of these, Early European Farmers, was putatively derived from West Eurasian Hunter Gatherers and “Basal Eurasians” which enjoyed isolated evolution distinct from all other out-of-Africa groups prior to genetic expansion into Europe. The Basal Eurasians appear to be the main candidate group associated with the earliest transition from West Eurasian hunter-gathering to agriculture^15^. Two subsequent studies seeking to identify the origin of these groups identified Yamnaya as a likely genetic origin in addition to contributing to Indo-European languages^16^,^17^. They differed on possible origins of Yamnaya, with one team, Haak *et al*., suggesting association possibly through the Caucasus^16^ and the other, Allentoft *et al*. denying it^17^. Interestingly, the Haak *et al*. study’s Y chromosome Yamnaya haplogroups were R1b1a or R1b1a2. Another recent study^38^ supports this result, though claims affinity with an “Armenian-like Near Eastern source.” Our study has identified the Caucasus refugium as the likely source for the J1 and J2 haplogroups that now dominate Southwest Asia, and previously appeared to mark the Neolithic Revolution’s expansion into Europe^39^. Yet, haplogroups J1 and J2 are distinctly lacking in the earlier Yamnaya samples.

The geographic separation of modern J haplogroups in Southwest Asia, and the prevalence of J2 but not J1 in the expansion into Europe is striking given their close origins^39^,^40^.

Population expansions from the identified refugia in this study, as demonstrated by archaeological tool culture impacts, served as sources for the demic diffusion of the Neolithic Revolution^2^,^11^ and the peopling of the Fertile Crescent. We further identify directions and timings of expansions and subsequent isolations which correlate well with both archaeological and climate data^2^,^4^,^7^,^9^,^10^,^11^,^27^,^34^,^36^,^41^. While aDNA studies have shown that genetic haplogroups in some populations elsewhere have been almost completely replaced over time^19^, the archaeological association of the expansions, and the reflection of those ancient expansions in the genetics of modern populations in the same region is notable. Additional aDNA studies in Southwest Asia will allow these population expansions to be tested further.

## Additional Information

**How to cite this article**: Platt, D. E. *et al*. Mapping Post-Glacial expansions: The Peopling of Southwest Asia. *Sci. Rep.* **7**, 40338; doi: 10.1038/srep40338 (2017).

**Publisher's note:** Springer Nature remains neutral with regard to jurisdictional claims in published maps and institutional affiliations.

## Supplementary Material

Supplementary Information

Supplementary Table 1

Supplementary Table 2

## Figures and Tables

**Table 1:** Times to most recent common ancestors for each haplogroup, measured in ka, for each region.

Region	*N*_*a*_	J	J1	J1e	J2	E1b1b1
Armenia	4.4 2.0–9.6	48 25–100	34 19–69	19 10–39	34 19–69	40 18–92
Caucasus*	4.8 1.0–9.3	41 21–82	26 14–52	—	26 14–52	83.3 42–174
Iran	7.5 4.1–14	59 30–124	35 19–68	—	35 19–68	36 19–73
Jordan	4.1 1.5–9.3	38 21–76	28 16–54	21 12–40	28 16–54	32 18–62
Kuwait	2.2 0.6–6	47 25–96	24 12–51	17 8.8–36	24 12–51	—
Lebanon	1.8 0.6–5.9	41 24–78	31 18–58	19 11–38	33 20–62	32 17–64
Palestinians	1.4 0.5–3.7	40 23–75	31 18–58	25 14–46	32 19–60	31 18–57
SE Turkey	5.2 2.4–11	52 27–109	29 15–60	16 8.5–34	33 18–66	38 19–80
Syria	3.2 0.9–8.1	48 28–88	35 21–64	26 15–46	35 21–64	29 16–60
Turkey	6.9 3.5–13	50 28–96	32 18–63	10 4.2–22	34 19–65	32 18–65
Algeria	3.6 0.9–10	64 33–135	27 13–60	—	29 14–62	—
Egypt	3.6 1.1–8.7	53 28–109	29 15–60	20 11–40	29 15–60	—
Iberia	8.6 5.1–15	65 33–135	—	—	30 16–61	—
Libya	2.0 0.7–5.2	38 20–81	26 13–56	16 7.7–36	—	—
Morocco	2.0 0.6–9.2	49 25–106	33 17–69	25 13–50	—	—
Tunisia	2.4 0.7–6.4	—	—	—	32 16–72	—

*N*_*a*_ is the ancestral population size (population size at time exponential expansion started), measured in 1,000’s.

^*^6 samples.
