# Supplementary material for: Mapping Post-Glacial expansions: The Peopling of Southwest Asia
Source: Sci Rep. 2017 Jan 6;7:40338. doi: 10.1038/srep40338 (PMC5216412; doi:10.1038/srep40338)
Supplement: Supplementary Information [file srep40338-s1.pdf]

Title: Mapping Post-Glacial expansions: The Peopling of Southwest Asia

Authors: Daniel E. Platt[1], Marc Haber[2,3], Magda Bou Dagher-Kharrat[4], Bouchra Douaihy[2], Georges Khazen[2], Maziar Ashrafian Bonab[5], Angélique Salloum[2], Francis Mouzaya[2], Donata Luiselli[6], Chris Tyler-Smith[3], Colin Renfrew[7], Elizabeth Matisoo-Smith[8], Pierre A. Zalloua[2,9,*],

Affiliations:

[1] Computational Biology Center, IBM TJ Watson Research Centre, Yorktown Hgts, NY, USA
[2] The Lebanese American University, Chouran, Beirut, Lebanon
[3] The Wellcome Trust Sanger Institute, Wellcome Trust Genome Campus, Hinxton, UK
[4] Département Sciences de la Vie et de la Terre, Université Saint-Joseph, Mkalles, Lebanon
[5] University of Portsmouth; School of Biological Sciences; Portsmouth PO1 2DY, UK.
[6] Department of Evolutionary Experimental Biology, Via Selmi, 340126 Bologna, Italy
[7] McDonald Institute for Archaeological Research. University of Cambridge, CB2 3ER, UK
[8] Department of Anatomy and Allan Wilson Centre for Molecular Ecology and Evolution, University of Otago, Dunedin, New Zealand
[9] Harvard School of Public Health, Boston, MA, USA

*Corresponding author: P A Zalloua, The Lebanese American University, School of Medicine Chouran, Beirut 1102 2801, Lebanon. Tel: +961 178 4408 Ext. 2855; Fax: +961 954 6090; E-mail: pierre.zalloua@lau.edu.lb

Short Title: Post-Glacial population expansions

Supplementary Information

Materials and Methods (supplemental)

Included studies

In addition, Y- Hg and haplotype data were also incorporated from prior studies (Table S1), including 727 newly genotyped samples from Armenia (ARM - 402 samples), Armenians originating in SE Turkey (TUR - 126), Bahrain (BAH - 40), Iraq (IRQ - 70), the Kingdom of Saudi Arabia (KSA - 7), Cyprus (CYP - 38) and Libya (LIB - 44)[1-15] . Haplogroup markers employed by the studies were catalogued (Table S2), from which most informative derived sets were constructed. The YHRD tree and ISOGG 2011 tree were compared, with YHRD being used as the base tree for derivation (also Table S2).

Y Haplogroup expansions

BATWING population splitting was applied to individual haplogroups drawn from both whole-population and haplogroup-specific studies. BATWING assumes a single-step mutation model, and coalescence, and it models population splitting by dividing the total parent population effective population size among the child populations. In practice, it has been noted that subsampling by haplogroups as opposed to random sampling appears to yield biased estimates of effective population sizes [16], revealing structure. We argue that the mutation and coalescence formulas are correct. Further we suggest that the “fixed, then expanding” population growth profile option, along with relatively wide confidence intervals, may provide a spline to the actual effective populations governing coalescence. Together, these would allow BATWING to model haplogroup expansions.

We justify using BATWING for this purpose as follows. BATWING constructs multiple trees and sampling population parameters (e.g. mutation rates and effective population sizes), computing likelihoods for each configuration employing Metropolis-Hastings Monte Carlo sampling, seeking likely regions of its range of parameters. After equilibration, BATWING presents various population-split tree hypotheses in proportion to their probabilities. Migration events produce minority trees if all haplogroups are combined: the modal trees reflect the dominating founding lineages represented in the populations [17]. In order to isolate and amplify the minority trees, we computed the population split times marked by specific haplogroups that likely marked the migration events. Coalescence within haplogroups are governed by the overall effective populations' sizes in which the evolution occurred; estimates of effective population size will reflect the coalescence rates marking the haplogroups' expansions.

Contour Map, PCA, MDS, BATWING Parameters, and other Details

Frequency (Figure S2) and variance (Figure S3) contour maps of the J1 and J2 haplogroups were constructed using the Kriging procedure [18] with Surfer 8 (Golden Software).

Principal component analysis (PCA) [19] was applied to relative haplogroup frequencies using prcomp [20,21] in R [21] . Kruskal's stress majorization multidimensional scaling [22] (MDS) was computed using isoMDS [20] in R [21] . The parameter set for BATWING [16] was that of Zhivotovsky's[23] mutation rate implemented in Xue et al. [24] Generally, burn-in cycles to achieve equilibration and convergence between pools ranged from 1.5 million to 3 million recorded samples. For Y-chromosome analysis, AMOVA[25] was provided in ARLEQUIN [26] . R_{ST} distances [27] were computed using ARLEQUIN [26] . Network 4.610 (Fluxus Engineering, Clare, U.K.) [28] was used to compute Reduced Median (RM) networks [29] using a reduction factor of 1.0, with no STR weighting, for haplogroups J1e, J*(xJ1e), J2a, J2b, and J2. Mantel distance

correlation tests [30] were employed to compute the distribution of correlations between two distance matrices under permutation.

PLINK [31] was used for genome-wide data management and quality control. A PCA was performed following SMARTPCA [32], iteratively removing outliers exceeding 2 standard errors, leaving 115 samples (15 Armenians, 20 Georgians, 12 Jordanians, 19 Lebanese, 10 Palestinians, 13 Saudi Arabians, 8 Syrian, 17 Turks, and 1 Yemeni), and plotted using R [21]. Genotypes of SNP j were assigned genotype scores of s_j with values of 0 for a homozygous major allele, 1 for heterozygous, and 2 for a homozygous minor allele. HWE was not assumed for estimations of mean and variance of score, so that $E(S) = p_{AB} + 2p_{BB}$, and $var(S) = p_{AB}(1 - p_{AB}) + 4p_{BB}(1 - p_{BB}) - 4p_{AB}p_{BB}$. In the case where the two haplotypes are in HWE, then $p_{AB} = 2p_B(1 - p_B)$, and the results reduce to $E(S) = 2p_B$, and $var(S) = 2p_B(1 - p_B)$, as expected.[33,34] The normalized sample SNP allele scores z_j were then defined as $z_j = \left(s_j - E(S)\right)/\sqrt{var(S)}$. Missing data are assigned a value of $z_j = 0$. Pairwise F_{ST} statistics were computed using[35]

$$F_{ST} = \frac{\pi_{Between} - \pi_{Within}}{\pi_{Between}}$$

where $\pi_{Between}$ and π_{Within} are the average number of pairwise differences between and within samples respectively.

PCA was computed as follows. The full data matrix, with the rows being N samples, and columns being D SNPs, is identified as d. This may be represented in terms of a singular value

decomposition as $d = usv^T$, where s is diagonal. It is assumed that the v^T is orthonormal, so that $v^T v = I$. Then $dd^T = usv^T vsu^T = us^2 u^T$. This implies u diagonalizes the symmetric matrix dd^T, and is therefore, itself, orthonormal. Given this, $v = d^T us^{-1}$, so that the samples may be represented in terms of an orthogonal basis that completely span the sampled subspace. Writing d in terms of sample vectors d_i of SNPs as normalized, $\sum_i d_i = 0$. The sum of squared distances is $d_{tot}^2 = \frac{1}{2}\sum_{ij}\left(d_i - d_j\right)^T\left(d_i - d_j\right) = \sum_i d_i^T d_i = tr(dd^T) = tr(us^2u^T) = tr(s^2u^Tu) = tr(s^2)$. Given a diagonal matrix P with 1's corresponding to the J largest eigenvalues in s, and zeros elsewhere, $P^2 = P$, and the matrix is idempotent (a projection matrix). Define $Q = I - P$. Then $d_{tot}^2 = tr\left((P+Q)s^2\right) = tr(Ps^2) + tr(Qs^2)$. The part $d_P = uPsv^T$ will capture $d_{tot,P}^2 = tr(Ps^2)$ of the total distance, so that a relatively small number of components of u may capture most of the genetic distance information simplifying the task of relating geographic associations between populations of data. The distribution of eigenvalues expected by chance has been described previously.[34]

In PCA, refugia would be expected to be marked by the largest genetic divergences between population clusters in a region reflecting the greatest periods of isolation. Generally, these correspond to the ancestral populations found by ADMIXTURE. Regions marked by larger divergences from others in PCA tend to be dominated by one or another ancestral population in ADMIXTURE. Pairwise F_{ST} statistics, computed using will also tend to be larger for more divergent autosomal samples. F_{ST} statistics have been a popular summary statistic, yielding information about equilibration as well as time dependence. However, they do not tend to yield information about likely distributions and uncertainties of time estimates. Cluster analysis, MDS, and other similar methods can yield alternative comparisons between F_{ST}, PCA and ADMIXTURE.

ADMIXTURE [36] was applied to the reduced set to identify ancestral populations and to compute ancestral F_{ST}s, and plotted using pophelper[37] implemented in R[21].

Divergence dates, assuming no admixture after splitting, were estimated from autosomal F_{ST} estimates using the relationship [38]

$$1 - F_{ST} = \left(1 - \frac{1}{2N_e}\right)^t$$

for isolation time t, fixation coefficient F_{ST}, effective population size N_e in the range for southwest Asians 7,006–9,505 [39], and a 29 year generation time [40]. In the presence of subsequent admixture, the time since a major split will tend to be under-estimated yielding a lower bound for a population split. Further, given the presence of variable effective populations (bottlenecks, etc), this time will tend to be underestimated.

Results

The Ward and Neighbor-Joining (NJ) cluster results for pairwise F_{ST}s estimated by the three leading MDS components (Figure S6) show the northern populations tending to isolate toward one end of the cluster, and southern populations isolating toward the other end, with Levantine and others in the middle. While the Ward's method clustering forced ultrametric lengths, as would be required for all samples to show the same evolutionary time, Neighbor-Joining forces edge lengths to be additive. F_{ST}s are expected to scale roughly with time, but depend on effective population sizes, migration and admixture, and variations in generation times. A predominately Palestinian branch includes a sub-branch with Syrians and Jordanians. Similarly, the NJ tree for Chromosome 2 also shows organization of northern and southern branches, with Levantine

branches including Syrians, Jordanians, Palestinians, and Lebanese, also strongly suggesting a Levantine refugium.

The unrooted NJ trees show strikingly varying branch lengths, and remarkably differentiated substructure within the major putative northern and southern refugia, as well as some suggestion of a Levantine refugium. The NJ tree for Chromosome 1 recapitulates the Ward's clustering result of the Syrian, Lebanese, and Palestinian expansion emerging from a northern branch, but the northern populations are also represented by two other branches that are not so closely related to each other. The remaining chromosomes show variations on these themes, reflecting the three ancestral populations. For those populations expanding from the north, they reveal a similar east/west split as identified for the J1/J2 expansions. We note the pairwise F_{ST} date estimates are much younger than the larger scale dates suggested by the ADMIXTURE derived F_{ST} date estimate. Agglomerative clustering [41] using Ward's method [42] and Neighbor Joining [43] were applied to the F_{ST} data.

Captions

Fig. S1. Geographic distribution of major Y chromosome haplogroups frequencies distribution in the Middle East, Arabia, North Africa, East Africa, and Europe from the current study and from the published data [1,2,10-12,14,39,44-50]. (Base map constructed from Map data: Google, Digital Globe, https://earth.google.com).

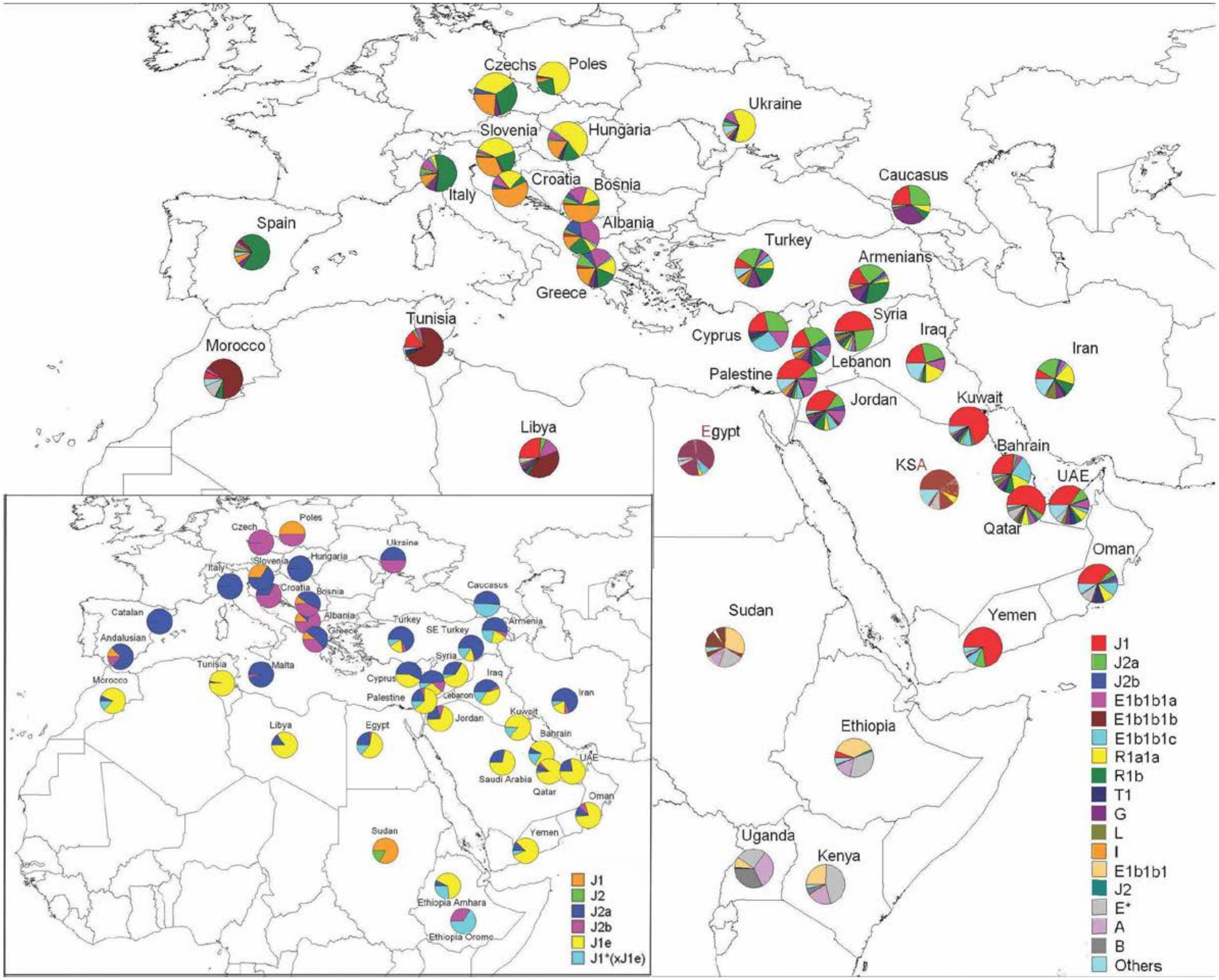

Czechs
Poles
Ukraine
Slovenia
Hungaria
Italy
Croatia
Bosnia
Albania
Greece
Spain
Caucasus
Turkey
Armenians
Tunisia
Morocco
Syria
Cyprus
Iraq
Iran
Lebanon
Palestine
Jordan
Kuwait
Libya
Egypt
Bahrain
KSA
UAE
Qatar
Oman
Sudan
Yemen
Ethiopia
Uganda
Kenya
J1
J2a
J2b
E1b1b1a
E1b1b1b
E1b1b1c
R1a1a
R1b
T1
G
L
I
E1b1b1
J2
E*
A
B
Others
Czech
Poles
Ukraine
Slovenia
Hungaria
Italy
Croatia
Bosnia
Caucasus
Catalan
Albania
Turkey
Armenia
Greece
SE Turkey
Andalusian
Tunisia
Malta
Syria
Morocco
Cyprus
Iraq
Iran
Palestine
Lebanon
Jordan
Kuwait
Libya
Egypt
Bahrain
UAE
Saudi Arabia
Qatar
Oman
Sudan
Yemen
Ethiopia Amhara
Ethiopia Oromo
J1
J2
J2a
J2b
J1e
J1*(xJ1e)

Fig. S2. Spatial frequency distribution of haplogroups **A)** J*(xJ2) and **B)** J2 throughout the Mediterranean basin. Contour maps constructed using the Kriging procedure with Surfer 8.09 (Golden Software, Inc. http://www.goldensoftware.com/products/surfer). Black dots show location of analyzed populations.

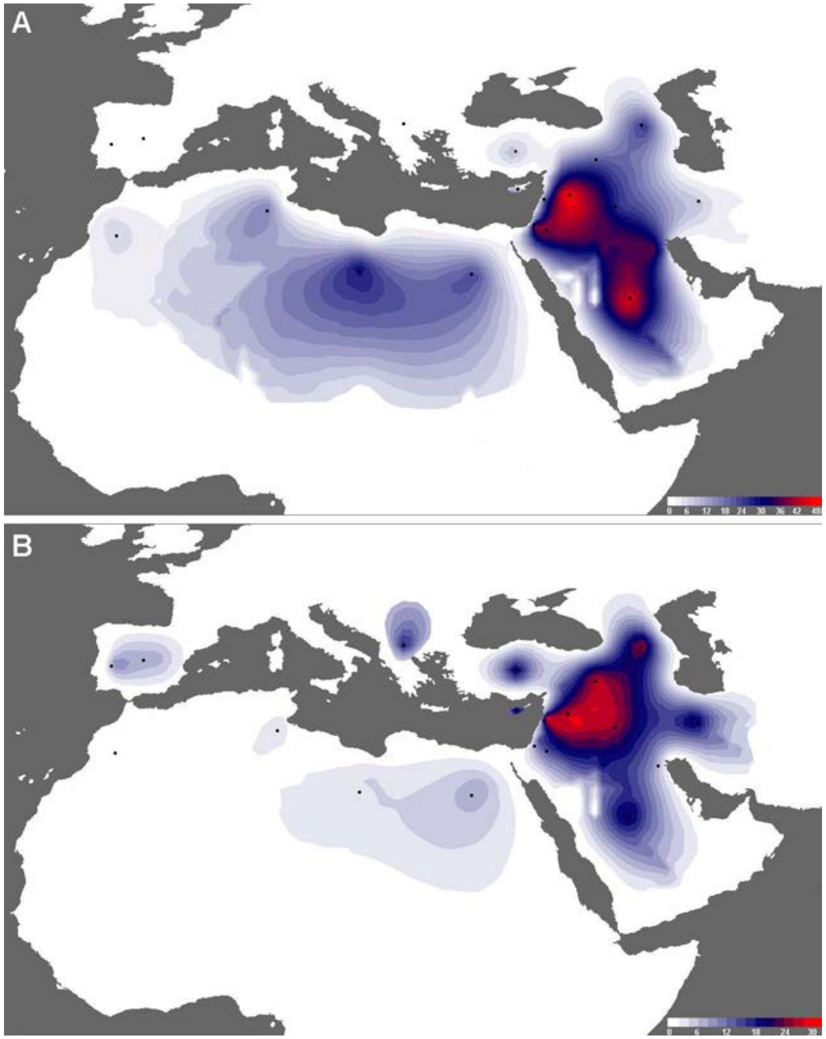
A
B

Fig. S3. Mean microsatellite variance distributions of haplogroups **A)** J*(xJ2) and **B)** J2 throughout the Mediterranean basin. Contour maps constructed using the Kriging procedure with Surfer 8.09 (Golden Software, Inc. http://www.goldensoftware.com/products/surfer). Black dots show location of analyzed populations.

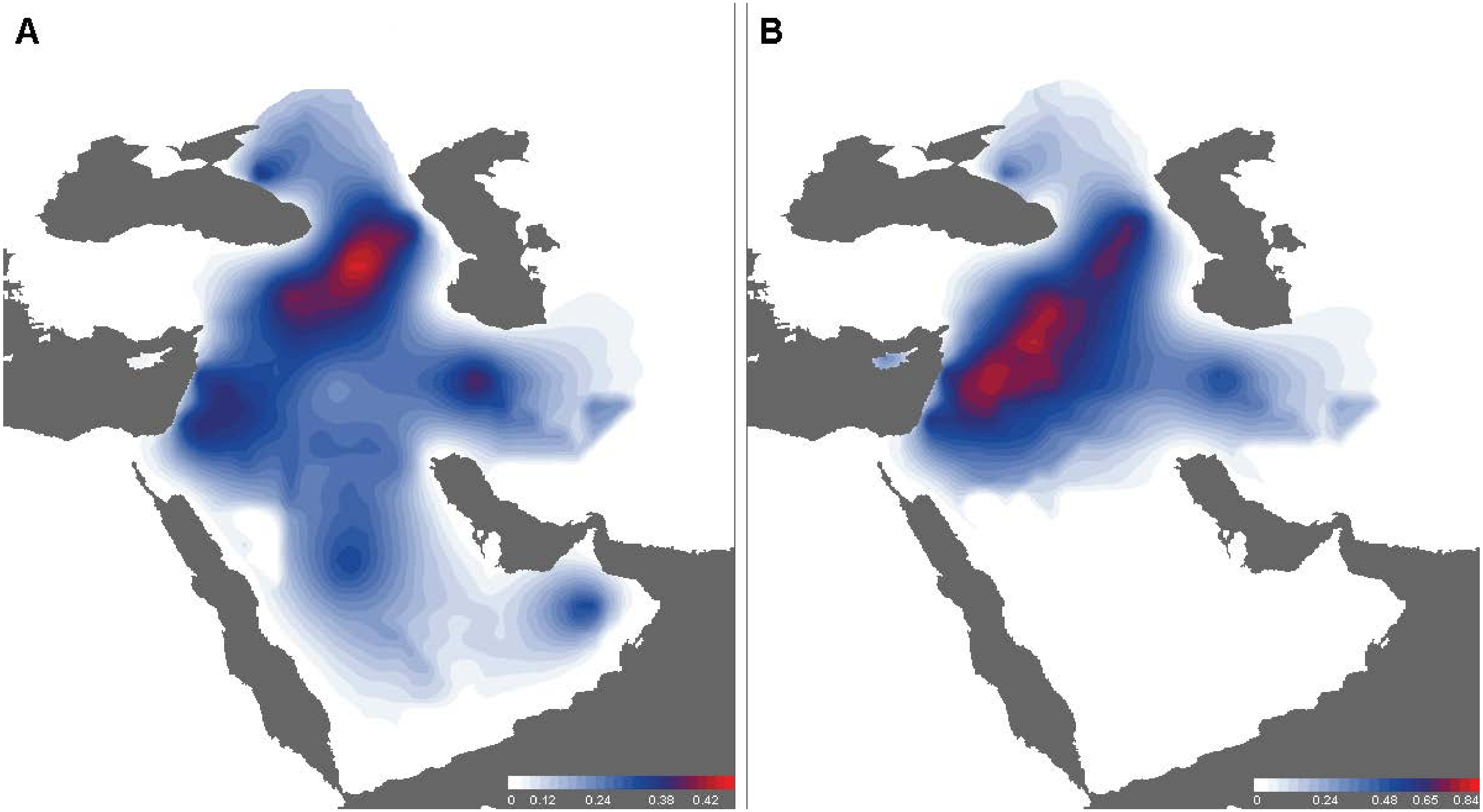

Fig. S4. A) Principal Component Analysis biplot computed using haplogroup frequencies on the most derived set of haplogroups over the Mediterranean Basin. **B)** Skree plot for the PCA analysis showing fraction of the variance explained by each component.

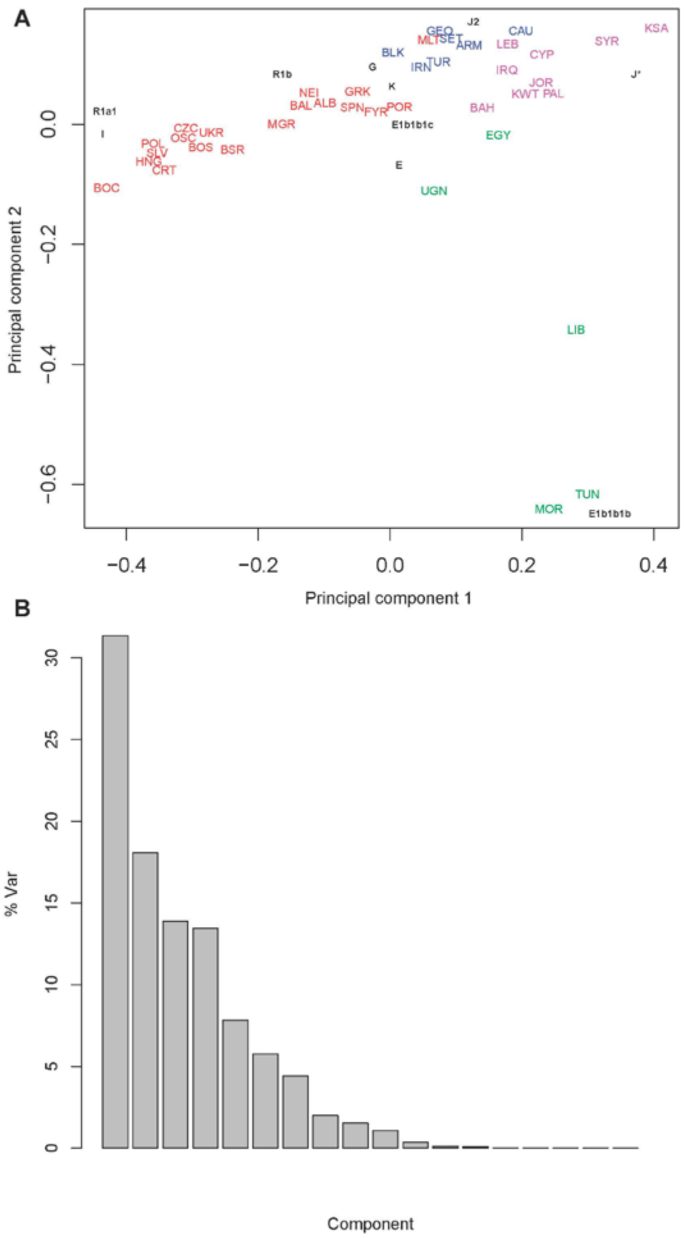
A
B
Principal component 1
Principal component 2
% Var
Component
R1a1
I
R1b
G
K
E1b1b1c
E
J2
J*
E1b1b1b
BOC
POL
SLV
HNG
CRT
CZC
OSC
UKR
BOS
BSR
MGR
NEI
BAL
ALB
GRK
SPN
FYR
POR
MLT
GEO
SET
ARM
BLK
IRN
TUR
CAU
LEB
CYP
IRQ
JOR
KWT
PAL
BAH
SYR
KSA
EGY
UGN
LIB
TUN
MOR

Fig. S5. Network of STR variation within J's subhaplogroups **A)** J2a, **B)** J2b **C)** J1e and **D)** J1*(xJ1e). Circles represent haplotypes defined by eight STRs; area is proportional to frequency, and color indicates the region of origin. Lines represent the mutational differences between haplotypes.

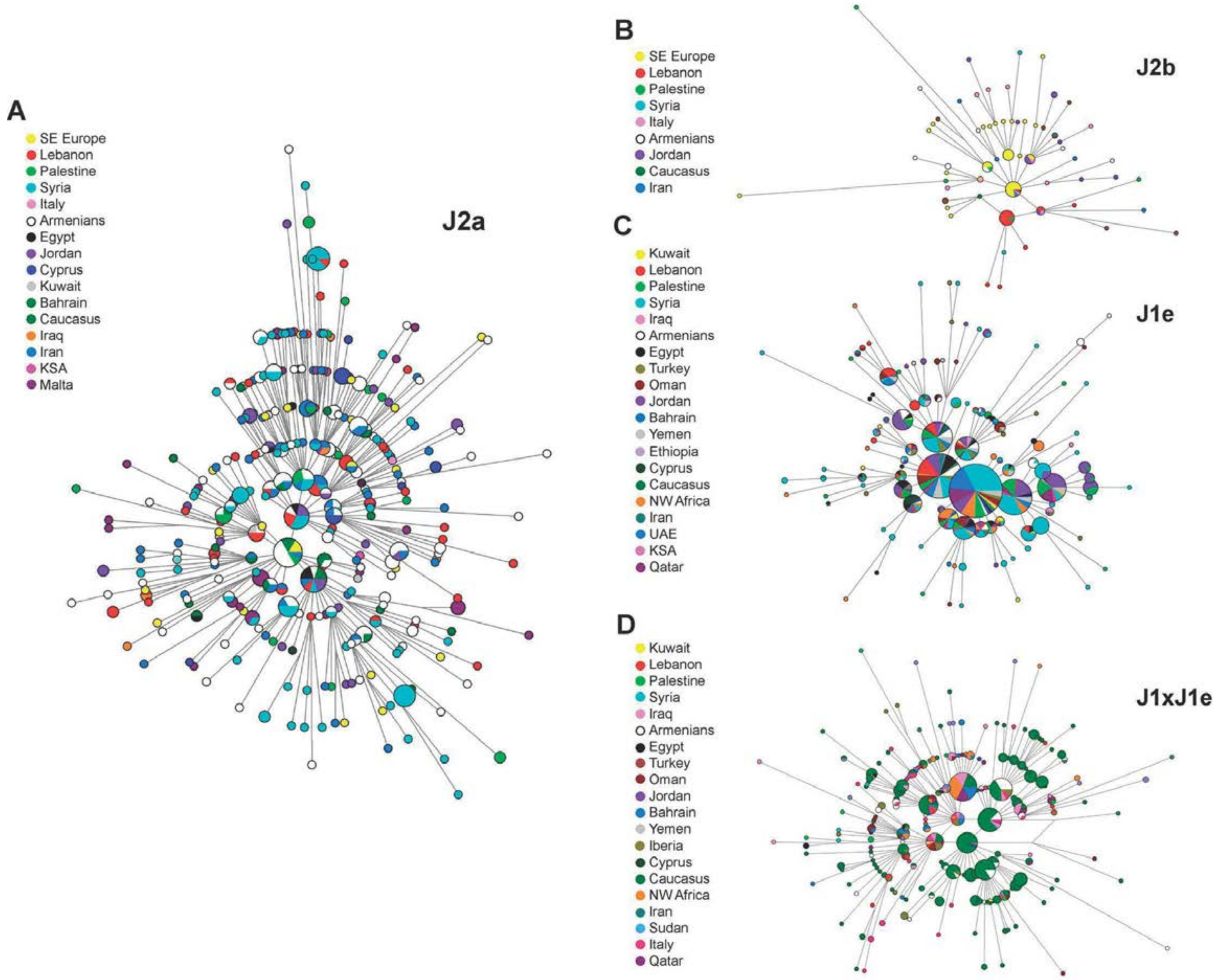

A
SE Europe
Lebanon
Palestine
Syria
Italy
Armenians
Egypt
Jordan
Cyprus
Kuwait
Bahrain
Caucasus
Iraq
Iran
KSA
Malta
J2a
B
SE Europe
Lebanon
Palestine
Syria
Italy
Armenians
Jordan
Caucasus
Iran
J2b
C
Kuwait
Lebanon
Palestine
Syria
Iraq
Armenians
Egypt
Turkey
Oman
Jordan
Bahrain
Yemen
Ethiopia
Cyprus
Caucasus
NW Africa
Iran
UAE
KSA
Qatar
J1e
D
Kuwait
Lebanon
Palestine
Syria
Iraq
Armenians
Egypt
Turkey
Oman
Jordan
Bahrain
Yemen
Iberia
Cyprus
Caucasus
NW Africa
Iran
Sudan
Italy
Qatar
J1xJ1e

Fig. S6. Pairwise FSTs between samples were reduced by MDS, with number of dimensions increased until range and distance histograms stabilized and reflected the initial data ranges. The three leading MDS components were used to construct distances clustered by Ward's method and by neighbor-joining. A) Chromosome 1, Ward's clustering; B) Chromosome 1, Neighbor- Joining; C) Chromosome 2, Ward's method; D) Chromosome 2, Neighbor-Joining.

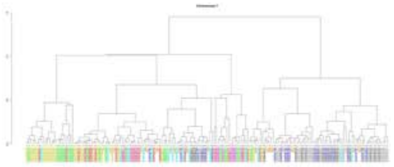

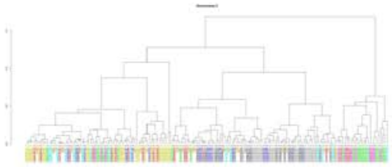

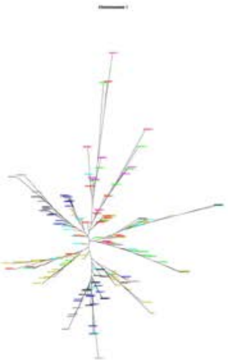

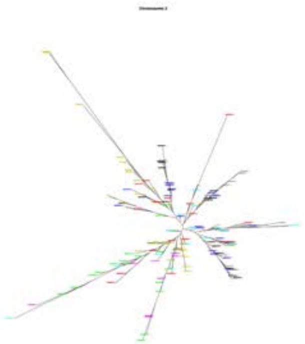

Table S1. Sample haplogroup and STR haplotype assignments for samples analyzed for this study.

Table S2. Comprehensive enumeration of Y chromosome markers, haplogroups according to YHRD and ISOGG 2011 phylogenies and nomenclatures, used to construct most informative derived sets of haplogroups.

References

1. de Filippo, C. et al. Y-chromosomal variation in sub-Saharan Africa: insights into the history of Niger-Congo groups. Molecular biology and evolution 28, 1255-1269, DOI:msq312 (2011).
2. Gomes, V., Sanchez-Diz, P., Amorim, A., Carracedo, A. & Gusmao, L. Digging deeper into East African human Y chromosome lineages. *Human genetics* **127**, 603-613, DOI:10.1007/s00439-010-0808-5 (2010).
3. Cinnioglu, C. *et al.* Excavating Y-chromosome haplotype strata in Anatolia. *Human genetics* **114**, 127-148, DOI:10.1007/s00439-003-1031-4 (2004).
4. Bosch, E. *et al.* Paternal and maternal lineages in the Balkans show a homogeneous landscape over linguistic barriers, except for the isolated Aromuns. *Ann Hum Genet* **70**, 459-487, DOI:10.1111/j.1469-1809.2005.00251.x (2006).
5. Balanovsky, O. *et al.* Parallel evolution of genes and languages in the Caucasus region. *Molecular biology and evolution* **28**, 2905-2920, DOI:10.1093/molbev/msr126 (2011).
6. Adams, S. M. *et al.* The genetic legacy of religious diversity and intolerance: paternal lineages of Christians, Jews, and Muslims in the Iberian Peninsula. *American journal of human genetics* **83**, 725-736, DOI:10.1016/j.ajhg.2008.11.007 (2008).
7. Ferri, G. *et al.* Male haplotypes and haplogroups differences between urban (Rimini) and rural area (Valmarecchia) in Romagna region (North Italy). *Forensic Science International* **175**, 250-255 (2008).
8. Ambrosio, B. *et al.* Searching the peopling of the Iberian Peninsula from the perspective of two Andalusian subpopulations: a study based on Y-chromosome haplogroups J and E. *Coll Antropol* **34**, 1215-1228 (2010).
9. Fadhlaoui-Zid, K. *et al.* Genetic structure of Tunisian ethnic groups revealed by paternal lineages. *Am J Phys Anthropol* **146**, 271-280, DOI:10.1002/ajpa.21581 (2011).
10. Battaglia, V. *et al.* Y-chromosomal evidence of the cultural diffusion of agriculture in Southeast Europe. *European journal of human genetics : EJHG* **17**, 820-830, DOI:10.1038/ejhg.2008.249 (2009).
11. Abu-Amero, K. K. *et al.* Saudi Arabian Y-Chromosome diversity and its relationship with nearby regions. *BMC Genet* **10**, 59, DOI:10.1186/1471-2156-10-59 (2009).
12. Cadenas, A. M., Zhivotovsky, L. A., Cavalli-Sforza, L. L., Underhill, P. A. & Herrera, R. J. Y-chromosome diversity characterizes the Gulf of Oman. *European journal of human genetics : EJHG* **16**, 374-386, DOI:10.1038/sj.ejhg.5201934 (2008).
13. Chiaroni, J. *et al.* The emergence of Y-chromosome haplogroup J1e among Arabic-speaking populations. *European journal of human genetics : EJHG* **18**, 348-353, DOI:10.1038/ejhg.2009.166 (2010).
14. Mohammad, T., Xue, Y., Evison, M. & Tyler-Smith, C. Genetic structure of nomadic Bedouin from Kuwait. *Heredity* **103**, 425-433, DOI:10.1038/hdy.2009.72 (2009).
15. Tofanelli, S. *et al.* J1-M267 Y lineage marks climate-driven pre-historical human displacements. *European journal of human genetics : EJHG* **17**, 1520-1524, DOI:10.1038/ejhg.2009.58 (2009).

16. Wilson, I., Balding, D. & Weale, M. Inferences from DNA Data: Population Histories, Evolutionary Processes and Forensic Probabilities. *Journal of the Royal Statistical Society: Series A (Statistics in Society)* **166**, 155-188 (2003).
17. Martinez-Cruz, B. *et al.* Evidence of Pre-Roman Tribal Genetic Structure in Basques from Uniparentally Inherited Markers. *Molecular biology and evolution*, DOI:10.1093/molbev/mss091 (2012).
18. Relethford, J. H. Geostatistics and spatial analysis in biological anthropology. *Am J Phys Anthropol* **136**, 1-10, DOI:10.1002/ajpa.20789 (2008).
19. Jolliffe, I. *Principal Components Analysis, Second Edition*. 2 edn, (Springer, 1986).
20. Venables, W. N. & Ripley, B. D. *Modern Applied Statistics with S*. (Springer-Verlag, 2002).
21. R Development Core Team. *R: A Language and Environment for Statistical Computing*. (R Foundation for Statistical Computing, 2011).
22. Kruskal, J. B. Multidimensional scaling by optimizing goodness of fit to a nonmetric hypothesis. *Psychometrika* **29**, 1-27 (1964).
23. Zhivotovsky, L. A. *et al.* The effective mutation rate at Y chromosome short tandem repeats, with application to human population-divergence time. *American journal of human genetics* **74**, 50-61, DOI:10.1086/380911 (2004).
24. Xue, Y. *et al.* Male demography in East Asia: a north-south contrast in human population expansion times. *Genetics* **172**, 2431-2439, DOI:genetics.105.054270 (2006).
25. Excoffier, L., Smouse, P. E. & Quattro, J. M. Analysis of molecular variance inferred from metric distances among DNA haplotypes: application to human mitochondrial DNA restriction data. *Genetics* **131**, 479-491 (1992).
26. Excoffier, L., Laval, G. & Schneider, S. Arlequin (version 3.0): An integrated software package for population genetics data analysis. *Evol Bioinform Online* **1**, 47-50 (2005).
27. Slatkin, M. A measure of population subdivision based on microsatellite allele frequencies. *Genetics* **139**, 457-462 (1995).
28. Bandelt, H. J., Forster, P., Sykes, B. C. & Richards, M. B. Mitochondrial portraits of human populations using median networks. *Genetics* **141**, 743-753 (1995).
29. Herrnstadt, C. *et al.* Reduced-median-network analysis of complete mitochondrial DNA coding-region sequences for the major African, Asian, and European haplogroups. *American journal of human genetics* **70**, 1152-1171, DOI:10.1086/339933 (2002).
30. Mantel, N. The detection of disease clustering and a generalized regression approach. *Cancer research* **27**, 209-220 (1967).
31. Purcell, S. *et al.* PLINK: a tool set for whole-genome association and population-based linkage analyses. *American journal of human genetics* **81**, 559-575, DOI:S0002-9297(07)61352-4 (2007).
32. Price, A. L. *et al.* Principal components analysis corrects for stratification in genome-wide association studies. *Nat Genet* **38**, 904-909, DOI:10.1038/ng1847 (2006).
33. Galinsky, K. J. *et al.* Fast Principal-Component Analysis Reveals Convergent Evolution of ADH1B in Europe and East Asia. *American journal of human genetics* **98**, 456-472, DOI:10.1016/j.ajhg.2015.12.022 (2016).
34. Patterson, N., Price, A. L. & Reich, D. Population structure and eigenanalysis. *PLoS Genet* **2**, e190, DOI:10.1371/journal.pgen.0020190 (2006).

35. Hudson, R. R., Slatkin, M. & Maddison, W. P. Estimation of levels of gene flow from DNA sequence data. *Genetics* **132**, 583-589 (1992).
36. Alexander, D. H., Novembre, J. & Lange, K. Fast model-based estimation of ancestry in unrelated individuals. *Genome Res* **19**, 1655-1664, DOI:10.1101/gr.094052.109 (2009).
37. Francis, R. M. pophelper: an r package and web app to analyse and visualize population structure. *Mol Ecol Resour*, DOI:10.1111/1755-0998.12509 (2016).
38. Holsinger, K. E. & Weir, B. S. Genetics in geographically structured populations: defining, estimating and interpreting F(ST). *Nat Rev Genet* **10**, 639-650, DOI:10.1038/nrg2611 (2009).
39. Zalloua, P. A. *et al.* Identifying genetic traces of historical expansions: Phoenician footprints in the Mediterranean. *The American Journal of Human Genetics* **83**, 633-642 (2008).
40. Fenner, J. N. Cross-cultural estimation of the human generation interval for use in genetics-based population divergence studies. *Am J Phys Anthropol* **128**, 415-423, DOI:10.1002/ajpa.20188 (2005).
41. Kaufman, L. & Rousseeuw, P. J. *Finding Groups in Data: An Introduction to Cluster Analysis*. (Wiley, 1990).
42. Ward, J. H. Hierarchical grouping to optimize an objective function. *Journal of the American Statistical Association* **58**, 236-244 (1963).
43. Saitou, N. & Nei, M. The neighbor-joining method: a new method for reconstructing phylogenetic trees. *Mol Biol Evol* **4**, 406-425 (1987).
44. Al-Zahery, N. *et al.* In search of the genetic footprints of Sumerians: a survey of Y-chromosome and mtDNA variation in the Marsh Arabs of Iraq. *BMC Evol Biol* **11**, 288, DOI:1471-2148-11-288 (2011).
45. El-Sibai, M. *et al.* Geographical structure of the Y-chromosomal genetic landscape of the Levant: a coastal-inland contrast. *Ann Hum Genet* **73**, 568-581, DOI:10.1111/j.1469-1809.2009.00538.x (2009).
46. Haber, M. *et al.* Influences of history, geography, and religion on genetic structure: the Maronites in Lebanon. *European journal of human genetics : EJHG* **19**, 334-340, DOI:10.1038/ejhg.2010.177 (2011).
47. Hassan, H. Y., Underhill, P. A., Cavalli-Sforza, L. L. & Ibrahim, M. E. Y-chromosome variation among Sudanese: restricted gene flow, concordance with language, geography, and history. *Am J Phys Anthropol* **137**, 316-323, DOI:10.1002/ajpa.20876 (2008).
48. Luis, J. R. *et al.* The Levant versus the Horn of Africa: evidence for bidirectional corridors of human migrations. *American journal of human genetics* **74**, 532-544, DOI:10.1086/382286 S0002-9297(07)61870-9 (2004).
49. Semino, O. *et al.* Origin, diffusion, and differentiation of Y-chromosome haplogroups E and J: inferences on the Neolithization of Europe and later migratory events in the Mediterranean area. *American journal of human genetics* **74**, 1023-1034, DOI:10.1086/386295 (2004).
50. Zalloua, P. A. *et al.* Y-chromosomal diversity in Lebanon is structured by recent historical events. *American journal of human genetics* **82**, 873-882, DOI:S0002-9297(08)00206-1 (2008).
